# Isolated dwarfism and sexual dimorphism in a mainland population of the greater short-horned lizard (*Phrynosoma hernandesi*) and the Great Plains toad (*Anaxyrus cognatus*)

**DOI:** 10.1371/journal.pone.0339275

**Published:** 2025-12-26

**Authors:** Megan E. Lahti, Leigh C. Latta, IV, Michael E. Pfrender, Edmund D. Brodie

**Affiliations:** Department of Biology, Utah State University, Logan, Utah, United States of America; CONICET: Consejo Nacional de Investigaciones Cientificas y Tecnicas, ARGENTINA

## Abstract

Body size is an important biological concept as it impacts nearly all aspects of an organism. In mainland systems, body size tends to show clinal variation; however, drastic body size shifts are typically limited to insular systems in correspondence with abrupt changes in climate patterns and resource availability, often in coordination with reduced genetic diversity. We investigate a rare occurrence of dwarfism and its influence on sexual size dimorphism (SSD) among two mainland species inhabiting the San Luis Valley (Valley) using museum and live specimens. The Valley population of the greater short-horned lizard (*Phrynosoma hernandesi*) is 33.7% smaller and the Valley population of the Great Plains toad (*Anaxyrus cognatus*) is 32.9% smaller than populations surrounding the Valley (Outside). The greater short-horned lizard exhibits female-biased SSD range wide, and female-biased SSD among Valley populations is maintained for head length and width but is inconsistent among test groups for radius, hand, and femur length. In the Great Plains toad, SSD is absent among museum specimens from the Outside population and live specimens the Valley population, but shows a weak presence in parotoid gland morphology among museum specimens from the Valley. Although the mechanisms underlying dwarfism and possible selective pressures associated with shifts in female-biased SSD are unknown, we present a rare instance of mainland dwarfism and its influence on SSD.

## Introduction

Body size is a fundamental biological trait that influences physiology, behavior, and ecology across all levels of biological organization (e.g., [1–4]). Dwarfism is one drastic form of body size shift, where isolation plays a key role, such as on islands [5,6]. The Island rule predicts that insular populations may shift toward dwarfism (or gigantism) in response to novel ecological conditions such as resource availability, competition, and predation [7]. Dwarfism may confer energetic advantages by reducing maintenance costs, improving heat exchange, and facilitating earlier reproduction [8,9]. In high-elevation or cold-climate systems, similar constraints on body size can arise from short growing seasons and extreme climate regimes [8,9]. Under such conditions, environmental isolation can act analogously to geographic isolation by restricting dispersal and reinforcing local adaptation. Genetic isolation can also drive dwarfism, often in association with reduced genetic diversity of the founding population (founder effect) and subsequent divergence via genetic drift [10]. Whether environmental or genetic, isolation is key to dwarfism because of its potential to release populations from traditional selection pressures while concurrently subjecting these populations to novel selective pressures and increased exposure to the effects of genetic drift or inbreeding [11].

In mainland populations, the possibility of isolation diminishes; both gene flow and environmental pressures are not typically varied enough at a local scale, geographic or temporal, for isolation to manifest. However, isolation, and subsequent shifts in body size, including sexual size dimorphism (SSD), is possible for species specialized to a local habitat or with low mobility across the landscape. Regardless of the mechanisms driving dwarfism, instances of localized dwarfism from mainland populations of a species are rare. We are aware of only one example of mainland dwarfism that is associated with a complex ecogeography. A dwarf population of *Testudo marginata* Schoepff, 1792 occurs along the Mani Peninsula (Peloponnese Peninsula, Greece), where adults are 25.4–29.5% smaller than the range wide average [12,13]. The dwarfed population lacks genetic distinction and is considered an ecotype of *T. marginata* [12,13]. However, individuals of intermediate size and morphologies are known, and it is hypothesized that reduced body size is driven by extreme environmental conditions [12]. Although uncommon, instances of mainland dwarfism offer valuable insights into understanding patterns in body size shifts, morphology, and SSD, from which mechanisms of selection can be investigated.

Within a species, differential selection on body size between females and males, in response to sexual and ecological selection, can lead to SSD [14]. These pressures can further influence variable survival, behavioral, and reproductive strategies between sexes. As with patterns in body size at the landscape scale, generalized rules have been made for SSD across taxa. According to Rensch’s rule, in taxa with female-biased SSD, the degree of SSD increases with decreasing body size [15]. Although Rensch’s rule is well supported in many larger taxa with male-biased SSD (wherein male-biased SSD increases with body size), its support among taxa with female-biased SSD remains limited. Instead, several studies demonstrate a reversal of Rensch’s rule in species with female-biased SSD (e.g., [16]). Patterns in SSD are important for understanding the evolutionary and ecological consequences of body size variation, particularly in relation to fecundity, population dynamics, and life-history trade-offs [17].

The San Luis Valley (Valley), Colorado is a high-elevation, cold desert in southern Colorado and northern New Mexico. The Valley supports a geographically isolated but genetically connected population of two terrestrial vertebrates: *Phrynosoma hernandesi* Girard, 1858 (greater short-horned lizard) and *Anaxyrus cognatus* Say, 1822 (Great Plains toad). Fossil evidence indicates that both species have occupied the Valley for nearly 1 million years, during which both populations had reduced body sizes and maintained gene flow with populations surrounding the Valley [18–21]. Both species are widely distributed across western North America but exhibit distinct clinal patterns of body size across their ranges [22]. *Phrynosoma hernandesi* reverses Bergmann’s rule, becoming smaller in size at higher latitudes. In contrast, *A. cognatus* shows no clear pattern of clinal variation in size. Similarly, female-biased SSD is consistent across the range of *P. hernandesi* and generally present in *A. cognatus*, although the degree and geographic extent of female bias in *A. cognatus*, particularly at northern and southern latitudes, remains unclear [17,23]. In this study, we investigate whether Valley populations of *P. hernandesi* and *A. cognatus* exhibit morphological patterns consistent with dwarfism and shifts in SSD. We test two hypotheses: (i) that populations within the Valley exhibit significantly reduced body size relative to surrounding populations, and (ii) that Valley populations exhibit greater female-biased SSD relative to surrounding populations. We predict that Valley populations of both species will exhibit smaller mean body sizes, consistent with localized dwarfism in a mainland system, and that the magnitude of female-biased SSD will increase as body size decreases, consistent with the predictions of Rensch’s rule.

## Materials and methods

### Study site and species

**Fig 1:**
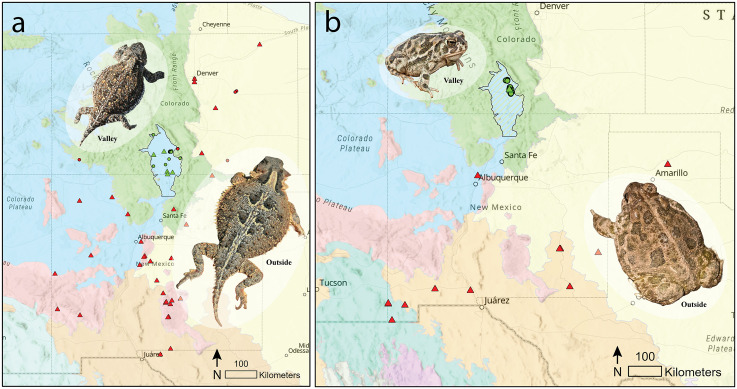
Distribution of *Phrynosoma hernandesi* (a) and *Anaxyrus cognatus* (b) museum (triangles) and live (circles) adults included in this study. The San Luis Valley (Valley) in south-central Colorado and north-central New Mexico is a rift valley at the southern end of the Rocky Mountains within the Western Cordillera (Northwestern Forested Mountains) ecoregion. It is North America’s largest high-elevation alpine desert, averaging 2,336 m elevation. The Valley belongs to the cold desert ecoregion and is surrounded by Temperate Prairie (Great Plains) ecoregion to the east and a continuation of the Cold Deserts (North American Deserts) ecoregion to the west. The Valley (green) population was compared against the surrounding population (Outside; red) to determine morphological variation among the Valley populations. Specimen images are proportionally scaled. Map was made in ArcGIS Pro 3.5.2 using Base Map: Terrain with Labels and Level II Ecoregions of North America feature layer (https://www.epa.gov/eco-research/ecoregions-north-america).

The San Luis Valley (Valley) is a 20,700 km^2^ rift valley in south-central Colorado and north-central New Mexico, formed by mountain ranges exceeding 4,267 m along the eastern (Sangre de Cristo Mountain range) and western boundaries (San Juan Mountain range) and is the origin for the Rio Grande River (Fig 1). The Valley is North America’s largest high-elevation alpine desert and averages 2,336 m elevation. The Valley belongs to the Cold Desert ecoregion [24]. Habitats occupied by Valley *P. hernandesi* populations include alluvial flats, sand dunes and sand sheets, and shrublands, while habitats occupied by Valley *A. cognatus* populations include salt flats and sand dunes and sand sheets. The Valley is immediately surrounded by South Central Semiarid Prairies (Great Plains) ecoregion to the east and a continuation of the Cold Deserts (Colorado Plateau) ecoregion to the south and west, while Upper Gila Mountains (San Francisco Plateau) and Warm Deserts (Chihuahua Desert) ecoregions occur further south [24]. Habitats occupied by Outside *P. hernandesi* populations include subalpine and conifer forests, montane woodland, semi-desert scrub and shrub-steppe, semi-arid and desert grassland and at elevations between 1,200–2,950 m. Habitats occupied by Outside *A. cognatus* populations include semi-arid and desert grassland and scrubland and at elevations between 900–1,600 m.

Adult *P. hernandesi* (greater short-horned lizard) and *A. cognatus* (Great Plains toad) were sampled in two geographically and ecologically distinct populations: (i) Valley population, limited to the San Luis Valley basin based upon geology and topography [25], and (ii) Outside population, which includes populations in closest proximity to and surrounding the San Luis Valley basin (Fig 1). Sampling localities within each species’ range are variable due to geographic distribution, sampling effort of natural populations, and existing adult museum specimens. Sampling of live specimens occurred on Federal and State protected land. We mapped localities for all specimens in ArcGIS Pro (Version 3.5.2, 2025, Esri, Redlands, CA, USA; Fig 1).

### Specimens

We measured museum and live *P. hernandesi* and *A. cognatus* specimens from populations within (Valley) and surrounding (Outside) the San Luis Valley (Fig 1). For *P. hernandesi*, measurements were obtained from 80 museum specimens (N = 39 females and 17 males from the Outside population; 12 females and 12 males from the Valley population) and 93 live specimens (N = 57 females and 36 males from the Valley population). For *A. cognatus*, measurements were obtained from 99 museum specimens (N = 15 females and 43 males from the Outside population; 17 females and 24 males from the Valley population) and 134 live specimens (N = 78 females and 56 males from the Valley population). Live specimens were collected by hand and measured between 2007 and 2009 under permit from Colorado (#08HP942), New Mexico (#3341), and Great Sand Dunes National Park (#GRSA-2008-SCI-001) following IACUC approved protocols (Utah State University #1315). We limited comparative morphometric analyses to adults to remove any effects of ontogenetic variation. Adult age class was assigned based upon minimum body size at reproductive maturity reported for outside populations [22] and determined for Valley populations through examination of reproductive status of both museum and live specimens. This conservative approach ensured that body size, which is a correlate of reproductive maturity, did not bias our determination of adult age classes or extent of dwarfism in Valley populations of *P. hernandesi* or *A. cognatus*. All measurements of both museum and field-sampled specimens were collected by a single investigator (MEL) to minimize measurement error.

External morphological features were chosen for measurement based on their ecological and behavioral importance for both species and limitations associated with live specimen measurements [26–28]. Measurements were taken on the right side of the body and to the nearest 0.1 mm using digital calipers (Fred V. Fowler Co., Inc., Newton, MA). A dissecting scope at 3X magnification using an ocular micrometer was used to measure *P. hernandesi* cranial horns due to their small size to the nearest 0.01 mm (S1 Table). Among *P. hernandesi*, 19 morphological features were measured in museum specimens and five morphological features were measured in live specimens (S1 Table). Among *A. cognatus*, 17 morphological features were measured using museum specimens and seven morphological features were measured using live specimens (S1 Table). Data from museum and live specimens were treated as separate data sets for both species to account for potential biases resulting from preservation-related shrinkage, variable measurement error, and temporal or geographic variation among populations [29,30].

### Analyses

The general approach we used to assess dwarfism and SSD in *P. hernandesi* and *A. cognatus* involved principal components analysis to generate multivariate ordinations and extract estimates of principal component 1 (PC1). Here, PC1 scores represent a metric that describes the multivariate body dimensions of an individual. After extraction of individual PC1 scores we then applied univariate analyses to individual traits and PC1 scores to test for dwarfism and SSD in both species.

Due to a combination of small sample sizes, non-normal distributions, and heterogeneous variances, we analyzed data with a combination of non-parametric approaches that accommodate these issues. Specifically, we used Kruskal-Wallis tests (KW), which are appropriate when distributions deviate from normality and/or if the variances are heterogeneous but the distributions (normal or not) have the same shape, and Welch’s ANOVA which is appropriate when distributions are normal but variances are heterogeneous. Both tests were applied to each trait and PC1 in each species in order to determine if our results were robust to the method of analysis. In every case, results from KW corroborated results from Welch’s ANOVA, with KW generating slightly higher estimates of p-values (S1 and S2 Files). Given the more conservative results from KW we present and interpret these results in the main text of this article.

For tests of dwarfism, our primary interest was to determine if there were population-level differences within each species (Outside vs. Valley) for each morphological trait and PC1. For tests of SSD, our interest was to determine if there were differences among the sexes (feale vs. male) within each population for each morphological trait and PC1. We note that KW precludes the inclusion of a covariate, so we are not testing allometric differences in these traits, only absolute differences in the size for each trait. Additionally, each trait was involved in up to four comparisons, and the total number of KW tests employed for each species was large (*P. hernandesi* – 66 total tests; *A. cognatus* – 62 total tests) which can lead to a multiple comparison problem. Using a simple Bonferroni correction to take into account the total number of KW tests applied to the data (128 tests) results in a new significance threshold of 3.91 x 10^−4^. Therefore, in order to err on the side of conservatism, we interpret results from our analyses with a p-value of greater than this threshold with caution. All analyses described subsequently were conducted in Program R v 4.3.3 [31].

### Tests for dwarfism

The presence of dwarfism in Valley populations of both *P. hernandesi* and *A. cognatus* was tested relative to populations surrounding the Valley using museum and live specimens. Historically, museum specimens and live specimens have been analyzed separately to account for morphological changes caused by preservation [29,30]. However, we did assess whether SVL in Valley specimens from the museum differed from live Valley specimens in both species using one-way ANOVA to determine if we should pool the datasets. Results from these analyses indicated that SVL in museum and live specimens of Valley *P. hernandesi* did differ (F = 6.2; df = 1; p = 0.017), while SVL in museum and live specimens of *A. cognatus* did not differ (F = 0.77; df = 1; p = 0.382). Given these contrasting results, we opted to employ the conventional approach of analyzing museum and live specimens separately for both species, although our interpretation of results for *A. cognatus* would not differ had we pooled the data. We used KW to test for an effect of population (Outside or Valley), regardless of sex, on individual traits and PC1 measured in museum specimens from each species. We then repeated these analyses for traits and PC1 measured in live specimens from each species. Estimates of PC1 were extracted for each individual from each species by applying PCA to the full data set for museum specimens, and then live specimens, resulting in a total of four PCAs (two for each species).

### Tests for sexual dimorphism

We used measurements from both museum and live specimens to determine the presence of SSD in both *P. hernandesi* and *A. cognatus*. However, because the degree of SSD may vary between Outside and Valley populations in each species, we subset the museum data into Outside and Valley subsets for each species. Data from live individuals was only collected from Valley populations for each species, so we used the full live dataset to test for SSD in each species. For each of the six datasets (Outside, Valley, and Live for each species) we used KW to test for an effect of sex (female or male) on individual traits and PC1. Estimates of PC1 were generated by applying PCA to each of the six datasets separately.

## Results

### Dwarfism in museum specimens of *P. hernandesi* and *A. cognatus*

**Fig 2:**
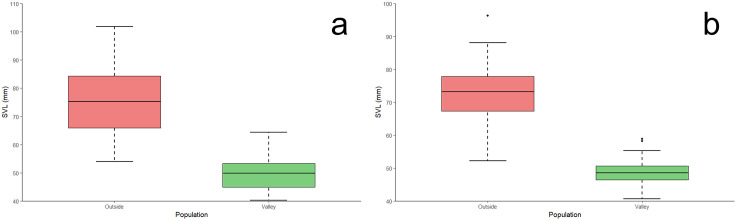
Body size (SVL) comparisons between *Phrynosoma hernandesi* (a) and *Anaxyrus cognatus* (b) museum specimens within (Valley) and surrounding (Outside) the San Luis Valley. Within the Valley, *P. hernandesi* were 33.7% smaller and *A. cognatus* are 32.9% smaller than populations outside the Valley (p < 0.0001).

**Fig 3:**
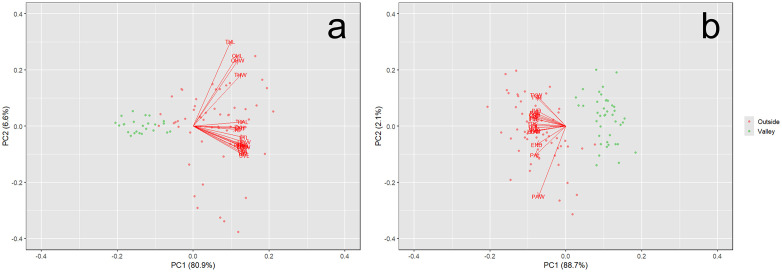
The PC plot for *Phrynosoma hernandesi* (a) and *Anaxyrus cognatus* (b) museum specimens within and surrounding the San Luis Valley. For both species, PC1 accounted for most of the variance (p < 0.0001).

Our results demonstrate that the Valley population of *P. hernandesi* exhibit reduced body size (SVL) relative to the Outside population (Figs 2a and 3a; S1 File). *Phrynosoma hernandesi* inside the Valley averaged 49.9 mm SVL (N = 24, median = 49.8 mm, range = 40.3–64.4 mm, SD = 6.67) while Outside lizards averaged 75.3 mm SVL (N = 56, median = 75.3 mm, range = 54.0–101.9 mm, SD = 12.11; SVL: χ ^2^ = 44.2, df = 1, p < 0.0001; PC1: χ ^2^ = 48.6, df = 1, p < 0.0001). Similarly, our analyses indicated the Valley population of *A. cognatus* exhibited reduced body size (SVL) relative to the Outside population (Figs 2b and 3b; S2 File). *Anaxyrus cognatus* inside the Valley averaged 48.7 mm SVL (N = 41, median = 48.6 mm, range = 40.6–58.9 mm, SD = 3.85) while Outside populations averaged 72.5 mm SVL (N = 59, median = 73.1 mm, range = 52.2–96.3 mm, SD = 8.04; SVL: χ ^2^ = 70.4, df = 1, p < 0.0001; PC1: χ ^2^ = 70.5, df = 1, p < 0.0001).

### Sexual dimorphism in *P. hernandesi* museum specimens

**Fig 4:**
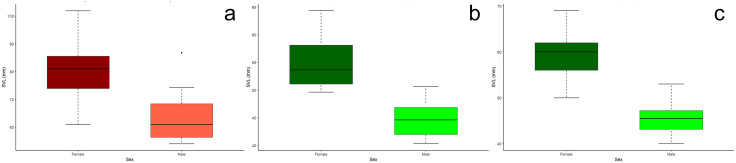
Body size (SVL) comparisons between female and male *Phrynosoma hernandesi* specimens indicated female-biased SSD in all populations. Among museum specimens from Outside **(a)** and Valley **(b)** populations, Valley females were 31.6% smaller and Valley males were 29.4% smaller than females and males outside the Valley, respectively. Among live specimens from the Valley population **(c)**, males were 23.4% smaller in body size than females.

Our results indicate SSD occurs in both Outside and Valley populations of *P. hernandesi*, with adult females displaying generally larger estimates of morphology than adult males outside and inside the Valley (Fig 4a and 4b; S1 File). Adult females were significantly larger than adult males among the Outside (χ ^2^ = 22.2, p < 0.0001) and Valley (χ ^2^ = 15.9, p < 0.0001) populations. Outside adult females averaged 80.4 mm SVL (N = 39, SD = 9.61, median = 81.0 mm, range = 61.0–101.9 mm) and Outside adult males averaged 63.5 mm SVL (N = 17, SD = 8.61, median = 61.0 mm, range = 54.0–86.8 mm). Valley adult females were an average of 31.6% smaller (N = 12, mean = 55.0 mm SVL, SD = 4.88, median = 53.7 mm, range = 49.6–64.4 mm) and Valley adult males were an average of 29.4% smaller (N = 12, mean = 44.8 mm SVL, SD = 3.56, median = 44.6 mm, range = 40.3–50.6 mm) than the corresponding sex from the Outside population.

**Fig 5:**
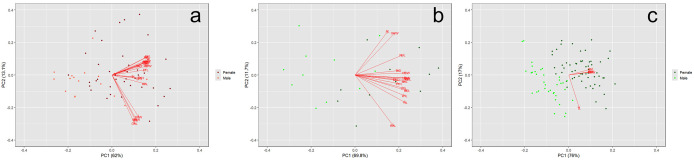
The PC plot for morphological features between female and male *Phrynosoma hernandesi* specimens. Collectively, the PC1 and PC2 axes represented 75.11% and 81.50% of the variance in morphology for museum specimens from the Outside **(a)** and Valley **(b)** populations, respectively. Among live specimens from the Valley population **(c)**, the PC1 and PC2 axes represented 93.00% of the variance in morphology.

There were significant differences among the additional 18 morphological features when comparing adult female and male *P. hernandesi* from Outside and Valley populations (Fig 5a and 5b; S1 File). Outside females had proportionally larger cranial (HDW, HDL, HSW) and forearm (RAL) features than Outside males (p < 0.0001) while Valley females had proportionally larger cranial (HDW, HDL) and hand (HAL) features compared to Valley males (p < 0.0001). While both populations showed significant differences in other traits at p < 0.001, we caution interpretation of these values due to an increase in Type I error associated with multiple comparisons.

### Sexual dimorphism in *P. hernandesi* live specimens

Our results indicate a difference in body size among female and male live *P. hernandesi* specimens within the Valley, with females being larger than males (χ ^2^ = 63.3, df = 1, p < 0.0001; Figs 4c and 5c; S1 File). Valley adult males were an average of 23.4% smaller (N = 36, mean = 45.4 mm SVL, SD = 3.28, median = 45.5 mm, range = 40.0–53.0 mm) than females (N = 57, mean = 59.3 mm SVL, SD = 5.26, median = 60.0 mm, range = 50.0–69.0 mm). Among live lizards in the Valley, females had a broader range of body sizes than males. Body size variation was 31.6% greater in females than males, and distribution of body size in both sexes was symmetrical (p > 0.05). In the live Valley population of *P. hernandesi*, female-biased SSD occurred in cranial (HDW, HDL) and limb (FEL) features (p < 0.0001).

### Sexual dimorphism in *A. cognatus* museum specimens

**Fig 6:**
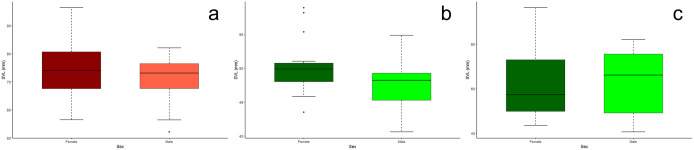
Body size (SVL) comparisons between female and male *Anaxyrus cognatus* specimens among museum specimens from Outside (a) and Valley (b) populations and live specimens from Valley (c) populations. Among museum specimens, Valley females were 32.6% smaller and Valley males were 33.9% smaller than females and males outside the Valley, respectively.

**Fig 7:**
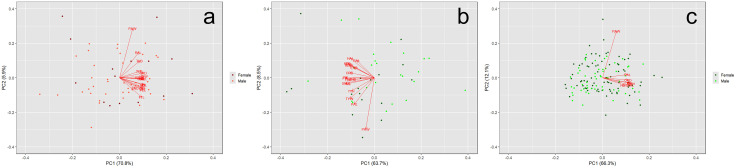
The PC plot for morphological features between female and male *Anaxyrus cognatus* specimens. Collectively, the PC1 and PC2 axes represented 76.73% and 72.17% of the variance in morphology for museum specimens from the Outside **(a)** and Valley **(b)** populations, respectively. Among live specimens from the Valley population **(c)**, the PC1 and PC2 axes represented 78.36% of the variance in morphology. Sexual size dimorphism was absent in the Outside population and minimally present within both museum and live specimens from the Valley population.

Our results indicated possible SSD in Valley populations of *A. cognatus*, but an absence in Outside populations (Figs 6a, 6b, 7a and 7b; S2 File). Valley females were larger than Valley males (χ ^2^ = 4.7, df = 1, p = 0.030), although this difference is not significant because it does not meet our conservative threshold of p < 0.0001 due to multiple comparisons. Outside females averaged 74.7 mm SVL (N = 15, SD = 11.27, median = 74.0, range = 56.5–96.3 mm) and males averaged 71.9 mm SVL (N = 43, SD = 6.62, median = 73.1, range = 52.2–82.0 mm). Valley females were an average of 32.6% smaller (N = 17, mean = 50.3 mm SVL, SD = 4.03, median = 49.9, range = 43.5–58.9 mm) and Valley males were an average of 33.9% smaller (N = 24, mean = 47.5 mm SVL, SD = 3.33, median = 48.3, range = 40.6–54.9 mm) than the corresponding sex from the Outside population.

Sexual-size dimorphism was absent from the 16 additional morphological features measured in both Outside and Valley populations (S2 File). While the Valley population showed significantly larger parotoid gland morphology (PAL, PAW) in females at p < 0.001, we caution interpretation of these values due to an increase in Type I error associated with multiple comparisons.

### Sexual dimorphism in *A. cognatus* live specimens

Live *A. cognatus* specimens in the Valley suggested SSD is absent for all traits including body size (SVL; Figs 6c and 7c, S2 File). Adult females averaged 50.1 mm SVL (N = 78, SD = 6.03, median = 49.7, range = 40.6–63.5 mm) and adult males averaged 48.7 mm SVL (N = 56, SD = 5.43, median = 47.5, range = 39.9–60.4 mm).

## Discussion

We report a rare example of dwarfism in two disparate species, *P. hernandesi* and *A. cognatus*, among populations within the San Luis Valley, Colorado. Valley wide, both species are over 30% smaller than surrounding populations, a finding in support of our first hypothesis that populations of both species within the Valley are significantly smaller in body size compared to Outside populations. Our second hypothesis that Valley populations exhibit greater female-biased SSD relative to surrounding populations is partially supported. Among Valley populations, female-biased SSD is maintained in *P. hernandesi* and may emerge in *A. cognatus* wherein patterns of emergence are inconsistent between museum and live specimens.

### Dwarfism

Evolutionary histories are often intertwined with geologic processes, particularly if the geologic formation serves as a reproductive barrier, and can provide insight into the history of the formation of a novel lineage. The San Luis Valley formed ca. 30 MYA and is North America’s largest high-elevation alpine desert ecosystem. Prior to its formation, the landscape was subject to several iterations of topographical reformation from the Laramide orogeny dating back to at least 300 MYA [32].

The fossil record indicates the presence of *P. hernandesi* and *A. cognatus* in the Valley at least 0.74 MYA and 0.84 MYA, respectively, and that populations of both species were dwarfed [33,34]. Combined fossil and pollen records indicate that within this 0.84 MA time frame, the Valley has experienced five oscillating climate cycles. The three warm-dry climate cycles were marked by sagebrush grassland habitat and high groundwater levels. Here, freezing duration was similar to the Valley’s current freezing duration (90–200 days), winters were relatively warm (normal minimum and mean daily temperatures in winter were probably greater than −5–0 °C), and annual temperatures were 3–8 °C warmer than today. The most recent climate cycle is cold-dry, having reduced precipitation and drier terrestrial conditions similar to the current cold high-desert environment. If either species showed periods of distributional shifts within the Valley, particularly during cold climate cycles, then it is unlikely to have been driven by these climate cycles. Both species have been consistently found in excavations from southeast New Mexico that correspond with cooler and wetter conditions than modern climate in those regions [35]. Additionally, both species are cold-tolerant and broadly distributed in higher latitudes and elevations [22,36–38].

Without age estimation, there is a potential for age structure to influence body size patterns in our study. However, several lines of evidence support the presence of dwarfism in both species rather than a sampling bias toward younger individuals. First, live specimens were sampled across four field seasons, and the mean body size in both species closely match those reported by Hahn [18] and Hammerson [39]. Second, our data set includes the largest reported individuals from the Valley populations of *P. hernandesi* (70.0 mm) and *A. cognatus* (63.5 mm), consistent with historical records [18,39]. Third, the smallest confirmed gravid *P. hernandesi* measured 56.0 mm SVL (unpubl. data), falling within the 30th percentile of body size for the Valley population of females, indicating that females reach sexual maturity at smaller sizes than Outside populations. Collectively, these findings support dwarfism as a stable, long-term morphological pattern restricted to Valley populations of both species.

*Phrynosoma hernandesi* is one of four species within the Tapaja crown clade which includes viviparity and reduced cranial horns [40]. Viviparity is associated with the cold climate hypothesis, and demonstrates the evolution of this clade at higher elevations and in colder climates. In the Tapaja clade, two species occur at extreme latitudes: *P. hernandesi* and *P. douglasii* Bell, 1829 (pygmy short-horned lizard) [41]. *Phrynosoma douglasii* is the smallest species among *Phrynosoma*; females average 53.1 mm SVL (range = 41–75 mm) and males average 41.5 mm SVL (range = 32–64 mm) (unpubl. data MEL). Comparatively, *P. douglasii* females are 10.4% and males are 8.6% smaller on average than dwarfed populations of *P. hernandesi*. Dwarfism could be proposed as an ancestral state in the Tapaja clade, as well as *A. cognatus*, wherein modern lineages have increased in body size and dwarf populations have retained an ancestral dwarf body size. However, fossil records for both the Tapaja clade and *A. cognatus* demonstrate body sizes similar to extant populations [42–50].

Although the origin of dwarfism in both species remains unknown, environmental pressures alone unlikely underly its emergence. Both species occur sympatrically with other herpetofauna in the Valley, yet only *P. hernandesi* and *A. cognatus* – two distantly related taxa – exhibit dwarfism [18,39]. The Valley’s cold-desert climate resembles that of southern Alberta, where both species reach their northern range limits. However, only *P. hernandesi* shows a distinct clinal reduction in body size with increasing latitude ([51, 52]; Parks Canada, unpubl. Data; Didiuk A, Canadian Wildlife Service, unpubl. data). Thus, cooler temperatures and shorter growing seasons may constrain growth in *P. hernandesi*, but this pattern is not evident in *A. cognatus*. Dietary limitations also appear unlikely, as Valley *P. hernandesi* consume a more diverse and abundant prey base than high-latitude populations [53]. Like *P. hernandesi*, *A. cognatus* is generally considered to be an opportunistic forager, with ontogenetic and temporal variation in diet [54,55]. However, dietary data for dwarf *A. cognatus* and other populations, particularly at northern latitudes, are needed to assess the influence of diet on body size [54]. Combined with fossil evidence indicating long-term persistence of dwarfism in Valley populations of both species, these patterns suggest that local environmental factors may reinforce but not cause dwarfism, particularly in *P. hernandesi*.

As with dwarfed populations of *T. marginata*, the importance of genetic data cannot be overlooked when determining whether a population represents a unique evolutionary lineage [12,21,56]. Genetic analyses of both species demonstrate that dwarfism can occur and persist in mainland systems without reproductive isolation or genetic divergence [19–21]. However, existing genetic data does not eliminate the possibility of a genetic basis for dwarfism. An interesting hypothesis that merits further investigation are the processes through which dwarfism established. A leading hypothesis might suggest that dwarfism arose separately from a novel mutation in both species, initially at a low frequency due to small effective population size or a bottleneck, spread via drift, and became fixed due to sexual selection associated with the small-male advantage hypothesis (discussed in next section). We encourage further investigation into the evolutionary history and underlying mechanisms of dwarfism to better understand the processes that allowed this rare phenomenon to occur.

### Sexual size dimorphism

Sexual size dimorphism has been a key topic of interest in biology because it spans such a great number of taxa, has profound evolutionary implications, and can be highly variable both within and among species and populations (e.g., [57,58]). Three generalized hypotheses explain mechanisms underlying SSD, with both *P. hernandesi* and, to a lesser-known degree– particularly at the latitudinal extremes of its range – *A. cognatus*, exhibiting female-biased SSD in body size across their ranges: (i) evolutionary constraints which result in a differential response between sexes to similar selective pressure(s), (ii) natural selection which results from differential responses to environmental competition when resources are limited and/or there is fecundity selection, and (iii) sexual selection which results in differential selection on one sex and a weaker but reciprocal response in the other sex [59]. These selective pressures inform Rensch’s rule, which explains the degree and patterns of SSD within and among lineages, and for which numerous studies have attempted to determine (e.g., [60,61]). However, generalized patterns of SSD are confounded by lower taxonomic comparisons and intraspecific variation due to underlying genetic variation, phylogenetic inertia, and variable environmental selective pressures (e.g., [62–64]). Patterns of SSD may also show intraspecific variation, as is seen in *P. platyrhinos* Girard, 1852 (desert horned lizard), which exhibits increased SSD in the northern extent of its range on account of males attaining smaller sizes at reproductive maturity [65].

Patterns of SSD vary considerably among vertebrate taxa. Male-biased SSD predominates in mammals and birds, largely in response to sexual selection, whereas female-biased SSD is more prevalent in insects, fishes, amphibians, and reptiles, largely in response to fecundity selection (e.g., [58]). In *P. hernandesi*, female-biased SSD is driven by selection for small male body size, known as the small-male advantage hypothesis [17,66]. In taxa with low population densities, including *Phrynosoma*, relative dispersal of females increases, competition among males decreases, and male reproductive success is determined by the number of females encountered. As a non-territorial species with minimal social structure, selection for smaller *P. hernandesi* males is additionally largely or fully released from the influence of selective pressures associated with the defense of territories or male-male competition [51]. In contrast to males, female body size might be a function of two competing factors: fecundity and minimum differential in body size from males required for reproductive capacity. If selective pressure for smaller males is stronger than selective pressures on female body size, then females would be expected to demonstrate reciprocal dwarfism. In *P. hernandesi*, fecundity positively correlates with body size among individuals, but not at the population level, likely due to low population densities and high mortality rates among neonates [17,51]. Dwarfed female *P. hernandesi* have smaller litters in both offspring count and size [53], yet dwarfed populations have successfully persisted within the Valley for nearly 1 million years. Dwarf females have a reduced range of body sizes, suggesting that dwarf populations may represent a minimum body size which would reflect limitations from environmental resources and/or life history.

In *P. hernandesi*, dwarfism corresponds with a shift in female-biased SSD, characterized by maintained head width and length, reduced head-shield width, and inconsistent limb proportions (radius, hand, and femur) among test groups. These patterns suggest that selection associated with feeding, via increased gape size in females, persist in dwarf populations and may reflect dietary niche partitioning or fecundity selection through greater energetic gain among gravid females [53,64,67]. Limb-length variation lacks a clear functional explanation but may reflect mobility limitations driven by selective pressures within the habitat rather than locomotor constraints imposed by dwarfism [68]. In *A. cognatus*, female-biased SSD is absent across all test groups, although parotoid morphology may show a weak female bias among dwarf populations. Given the marginal significance of some traits and limited sample sizes, broader geographic analyses are needed to assess how body size, and particularly dwarfism, influences SSD and its mechanisms. For example, shifts in female-biased SSD among dwarf populations may reflect developmental constraints or novel selective pressures, such as selection for reduced limbs in the Valley’s high-elevation, cold-desert environment, as predicted by Allen’s rule [69]. These findings provide initial evidence that dwarfism influences SSD and emphasizes the need for expanded analyses incorporating evolutionary and ecological processes.

The types and degree of selective pressures influencing SSD in *A. cognatus* remains poorly known, although the fecundity advantage hypothesis explains SSD in most anurans [70]. Inconsistencies in the emergence of SSD in museum and live *A. cognatus* specimens may be confounded by variation related to specimen preservation and storage, especially for body parts with soft tissues (e.g., [29]), measurement errors associated with difficulty in handling live specimens, or sample sizes confounded by developmental plasticity [71]. Further investigation of morphological features between museum and live specimens is needed to clarify patterns in the emergence of SSD in dwarfed populations of *A. cognatus*.

Body size and SSD vary across geographic and environmental gradients in many species, as climate, habitat diversity, predators, and competition can be dynamic and variable, even at a local spatial and temporal scale [72]. Sometimes, responses to ecological variation, not sexual selection, are more influencing to SSD than genetics and require further field studies to discern [73]. Similarly, the influence of environmental selective pressures on morphology can be exacerbated in species with low dispersal rates, such as in *P. hernandesi* and *A. cognatus*, and when delimited by geologic and ecological barriers [17,74]. Therefore, patterns in body size and SSD are best understood within evolutionary, environmental, and life history contexts. Our study demonstrates dwarfism and subsequent shifts in SSD among two species within a mainland system. Determining whether these patterns result from adaptive responses, genetic drift, or developmental constraints is important to further our understanding of body size evolution and SSD.

## Supporting information

S1 Table Morphological features measured in both museum and live specimens for *P. hernandesi* and *A. cognatus.* (DOCX)

S1 File Source data, descriptive statistics, and statistical analyses for morphological traits in museum and live *P. hernandesi* specimens. All measurements were taken using digital calipers except OHL, OHW, THL, THW, which were measured using a dissecting microscope (3X) with an ocular micrometer. For all traits with the exception of SVL, we caution interpretation of p-values > 10^−4^ due an increase in Type 1 error associated with multiple comparisons.
(XLSX)

S2 File Source data, descriptive statistics, and statistical analyses for morphological traits in museum and live *A. cognatus* specimens. All measurements were taken using digital calipers except TUB, which was measured using a dissecting microscope (3X) with an ocular micrometer. For all traits with the exception of SVL, we caution interpretation of p-values > 10^−4^ due an increase in Type 1 error associated with multiple comparisons.
(XLSX)
