# Supplementary material for: Isolated dwarfism and sexual dimorphism in a mainland population of the greater short-horned lizard (Phrynosoma hernandesi) and the Great Plains toad (Anaxyrus cognatus)
Source: PLoS One. 2025 Dec 26;20(12):e0339275. doi: 10.1371/journal.pone.0339275 (PMC12742731; doi:10.1371/journal.pone.0339275)
Supplement: S1 Table — (DOCX) [file pone.0339275.s001.docx]

**Supporting Table 1.** Morphological features measured in both museum and live specimens for *P. hernandesi* and *A. cognatus*.

| **Measurement** | **Code** | **Definition**  (a = *P. hernandesi*, b = *A. cognatus*) | ***P. hernandesi*** | | ***A. cognatus*** | |
| --- | --- | --- | --- | --- | --- | --- |
|  |  |  | **Museum** | **Live** | **Museum** | **Live** |
| Body size | SVL | snout tip to the vent | X | X | X | X |
| Tail length | TL | vent to the tail tip | X | X |  |  |
| Head width | HDW | distance between the angles (corners) of the lower jaws (widest part of the head) | X |  | X | X |
| Head length | HDL | a) snout tip to the rostral scale;  b) snout tip to the posterior skull | X | X | X |  |
| Head shield width | HSW | distance between the distal edges of the anterior superciliary spines | X | X |  |  |
| Occipital horn length | OHL | base to the tip of the medial plane of the occipital horn | X |  |  |  |
| Occipital horn width | OHW | widest point between medial and lateral base of the occipital horn | X |  |  |  |
| Temporal horn length | THL | base to the tip of the medial plane of the temporal horn | X |  |  |  |
| Temporal horn width | THW | widest point between medial and lateral base of the third temporal horn | X |  |  |  |
| Eye-nostril distance | END | posterior orbit to the nasal opening | X |  | X |  |
| Internsal distance | IND | the distance between the two nasal openings | X |  | X |  |
| Tympanum height | TYH | medial (maximum) height of the tympanum membrane surface | X |  | X |  |
| Tympanum width | TYW | medial (maximum) width of the tympanum membrane surface |  |  | X |  |
| Humerus length | HUL | apex of the glenohumoral joint to the distal end of the radiocarpal joint of the extended wrist | X |  | X |  |
| Radius length | RAL | proximal end of the radiohumeral joint to the distal end of the radiocarpal joint of the extended wrist | X |  | X |  |
| Hand length | HAL | proximal end of the radiocarpal joint of the extended wrist to the tip of the last scale on the fourth (longest) toe | X |  | X |  |
| Femur length | FEL | half the distance between both tibiofemoral joints when the legs are positioned 180˚ from each other in the same plane | X | X | X | X |
| Tibia length | TIL | proximal end of the tibiofemoral joint to the distal end of the subtalar joint | X |  | X |  |
| Foot length | FTL | a) proximal end of the subtalar joint to the last scale on the fourth (longest) toe;  b) proximal end of the subtalar joint to the tip of the fourth (longest) toe | X |  | X |  |
| Longest hindfoot toe | LHT | interdigital webbing on the medial side of the fourth (longest) toe to the last scale on the fourth toe | X |  |  |  |
| Orbit diameter | ORD | distance between the anterior and posterior corners of the ocular orbit |  |  | X | X |
| Parotoid gland length | PAL | length of the parotoid gland along the midline |  |  | X | X |
| Parotiod gland width | PAW | width of the parotoid gland at the midline |  |  | X | X |
| Tubercle length | TUB | proximal to distal base of the tubercle |  |  | X |  |
| Mass | - | mass in grams (g) |  |  |  | X |
